# Caught in the web: Spider web architecture affects prey specialization and spider–prey stoichiometric relationships

**DOI:** 10.1002/ece3.4028

**Published:** 2018-05-30

**Authors:** Lorraine Ludwig, Matthew A. Barbour, Jennifer Guevara, Leticia Avilés, Angélica L. González

**Affiliations:** ^1^ Department of Zoology Biodiversity Research Centre University of British Columbia BC Canada; ^2^ Universidad Regional Amazónica IKIAM Tena Napo Ecuador; ^3^ Department of Biology Center for Computational and Integrative Biology Rutgers University Camden NJ USA; ^4^ Department of Evolutionary Biology and Environmental Studies University of Zurich Switzerland

**Keywords:** ecological stoichiometry, food webs, nitrogen, phosphorus, predator–prey interactions, spider webs, threshold elemental ratio

## Abstract

Quantitative approaches to predator–prey interactions are central to understanding the structure of food webs and their dynamics. Different predatory strategies may influence the occurrence and strength of trophic interactions likely affecting the rates and magnitudes of energy and nutrient transfer between trophic levels and stoichiometry of predator–prey interactions. Here, we used spider–prey interactions as a model system to investigate whether different spider web architectures—orb, tangle, and sheet‐tangle—affect the composition and diet breadth of spiders and whether these, in turn, influence stoichiometric relationships between spiders and their prey. Our results showed that web architecture partially affects the richness and composition of the prey captured by spiders. Tangle‐web spiders were specialists, capturing a restricted subset of the prey community (primarily Diptera), whereas orb and sheet‐tangle web spiders were generalists, capturing a broader range of prey types. We also observed elemental imbalances between spiders and their prey. In general, spiders had higher requirements for both nitrogen (N) and phosphorus (P) than those provided by their prey even after accounting for prey biomass. Larger P imbalances for tangle‐web spiders than for orb and sheet‐tangle web spiders suggest that trophic specialization may impose strong elemental constraints for these predators unless they display behavioral or physiological mechanisms to cope with nutrient limitation. Our findings suggest that integrating quantitative analysis of species interactions with elemental stoichiometry can help to better understand the occurrence of stoichiometric imbalances in predator–prey interactions.

## 1 INTRODUCTION

Biological communities are composed of networks of trophic interactions, whose structure plays a major role in shaping the dynamics and functioning of ecological systems (Montoya, Pimm, & Solé, 2006; Olff et al., 2009; Pimm, 1982). Such networks, which look at the degree to which predators feed upon a prey community, can be used to observe both qualitative properties—which species are interacting—or quantitative measures that consider the strength of the species interactions (Ings et al., 2009; Williams & Martinez, 2000). While qualitative descriptors are based on the presence or absence of trophic links, quantitative metrics can include bioenergetics estimations. These bioenergetics estimates may reveal new aspects of food web structure, as they quantify the transfer of energy and matter among organisms within a community (DeAngelis, 1992; Link, Stockhausen, & Methratta, 2005). To date, however, relatively few studies have used bioenergetics approaches to understand consumer–resource interactions (Cohen, Briand, & Newman,^1990^; Lindemann, 1942; Reuman & Cohen, 2005).

Recent advances in food web analysis integrate both energy and matter transfers among species as quantitative estimators of food web structure and function (Hall, 2009; Olff et al., 2009; Sterner & Elser, 2002; Sterner, Elser, Chrzanowski, Schampel, & George, 1996). All organisms interacting in food webs share a biochemical makeup of predominantly carbon (C), nitrogen (N), and phosphorus (P), with other chemical elements (Elser et al., 2000; Sterner & Elser, 2002). Organisms of different taxonomic groups, body sizes, and feeding modes, however, differ widely in the proportion of each element in their biomass (Fagan et al., 2002; González, Fariña, Kay, Pinto, & Marquet, 2011; Lemoine, Giery, & Burkepile, 2014; Woods et al., 2004). According to the theory of ecological stoichiometry (Sterner & Elser, 2002), differences in the elemental composition between trophic levels generate elemental imbalances between consumers and their resources, which may impose strong constraints on trophic interactions (Mulder et al., 2013; Sterner & Elser, 2002).

Recent findings suggest that predators may face elemental imbalances between their nutrient demands and the supply of those nutrients by their prey (Fagan & Denno, 2004; Matsumura et al., 2004; Okuyama, 2008). As insect prey species typically vary in their body elemental content (Fagan et al., 2002; González et al., 2011), specialization can increase the strength of trophic interactions, but may also impose constrains on the transfer of energy and matter (Sanders, Vogel, & Knop, 2015; Shurin, Gruner, & Hillebrand, 2006). For example, prey specialization may affect interaction strengths if N‐rich consumers specialized on poor‐quality resources have to consume several individuals (i.e., compensatory feeding) of the prey species to supply their needs for N (Fagan & Denno, 2004; Huberty & Denno, 2006; Siuda & Dam, 2010). In contrast, generalist consumers may rely on food selection to meet their nutritional demands (Huberty & Denno, 2006). Therefore, the integration between stoichiometry and food web analysis can allow us to trace the pathways and constraints of energy and matter within communities, providing a strong link between food web structure and function (Mulder et al., 2013; Sterner & Elser, 2002; Woodward et al., 2005).

**Figure 1:**
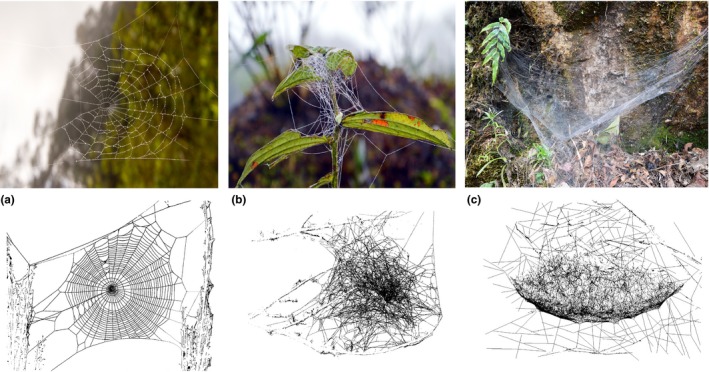
Diversity of web architectures; (a) Orb web, (b) Tangle web, and (c) Sheet‐tangle web. Photographs by A.L. González and drawings by J. Solar

Web‐building spiders are outstanding model systems for the study of trophic interactions because actual predator–prey interactions are relatively easy to quantify. Web architecture differs greatly among spider species, ranging from the commonly known orb construction, to three‐dimensional tangles with crossing lines of silk, such as tangle webs, and those with a dense basal sheet referred to as sheet‐tangle webs (Figure 1) (Savory, 1960). These differences in the architecture of the webs may result in different types of prey being captured (Blamires, 2010; Guevara & Avilés, 2009; Sanders et al., 2015). Although the nutritional content of captured prey is beyond the control of the spider (Mayntz, Raubenheimer, Salomon, Toft, & Simpson, 2005), web architecture likely affects the energy pathways and the stoichiometry of spider–prey interactions via selective capture and feeding (Mayntz, Toft, & Vollrath, 2009; Schmidt et al., 2012).

In this study, we combined prey capture surveys of web‐building spiders and consumer–resource elemental stoichiometry to explicitly examine the structure of spider–prey food webs on the basis of prey frequency, biomass, and the stoichiometry of trophic interactions. Specifically, we addressed the following questions: (1) How does spider web architecture influence prey species richness and composition (i.e., the relative frequency and biomass of prey types)? and (2) Does web architecture influence the stoichiometry of spider–prey interactions? We hypothesized that if web architecture influences spider–prey interactions, then spiders that build webs of different architectures will differ in the prey communities they capture, resulting in relative prey capture variability and degree of generalist trophic behavior as a function of web type. Furthermore, as web‐building spiders typically face prey heterogeneity in availability (i.e., abundance, variability, and predictability) and range of prey types (Scharf, Lubin, & Ovadia, 2011), and prey taxa can vary significantly in their nutrient composition (Fagan et al., 2002; González et al., 2011), hunting modes associated to different web architectures may affect the stoichiometry of spider–prey interactions. As generalist predators feed on a wide variety of prey, they may be able to selectively combine prey in their diets to balance their nutritional needs (Mayntz & Toft, 2001; Oelbermann & Scheu, 2002). In contrast, specialist consumers being restricted to a narrower range of prey types may face nutrient limitation if a single prey taxon does not satisfy the nutritional needs of the consumer (Westoby, 1978). In such a case, we expect that specialization will result in spiders with greater elemental imbalances than those faced by generalist spiders. Alternatively, more specialist predators may benefit from feeding on specific prey if the single prey is of optimal nutrient content. In the latter case, foraging theory predicts that selectivity should be favored if there are concrete nutritional benefits derived from feeding on specific prey types (Stephens & Krebs, 1986). Based on this, we expect that specialization will result in spiders with weaker elemental imbalances than generalist spiders.

## 2 METHODS

### 2.1 Study site

We conducted this study at the Jatun Sacha Biological Reserve (1°42′0″ S, 77°36′36″ W) in the Napo province of Ecuador, between July and August 2014. The reserve consists of 2,200 ha of primary and about 750 ha of secondary Amazon rainforest. The annual mean temperature at Jatun Sacha is 25°C, with yearly rainfall of 5,000 mm (Guevara & Avilés, 2009).

This study focused on 11 common spider species with three main types of web architectures. Orbs (two‐dimensional webs with no barrier) were built by four different species: two species of Araneidae (*Cyclosa* sp. and *Eriophora* sp.), an Uloboridae (*Uloborus* sp.), and a colonial Tetragnathidae species (*Leucauge* sp.). Tangles (three‐dimensional webs with no dense sheet) were built by species from the family Theridiidae (*Parasteatoda* sp., *Theridion* sp., and *Chrysso* sp.) and by the web‐building Pisaurid genus *Architis*. Lastly, sheet‐tangles (three‐dimensional webs with a dense basal or central sheet) were built by two social species from the family Theridiidae (*Anelosimus domingo* and *Anelosimus eximius*), and two solitary species from the families Araneidae (*Kapogea sexnotata*) and Lycosidae (*Aglaoctenus castaneus*) (Figure 1). Social spiders live in large communal webs that can host thousands of individuals, which cooperate in brood care, web maintenance, and prey capture.

### 2.2 Prey capture observations

Prey capture data were collected by the same two observers over seven days by recording the prey caught in spider webs found along a trail traversing a ~2 km^2^ area of secondary rainforest in the Jatun Sacha reserve. The trails were chosen based on the presence and abundance of spiders, their accessibility, and little to no foot traffic to ensure that spiders would not be disturbed. Webs of different architectures were tagged and assigned an identification code on the first day of observation. Two orb weavers whose webs disappeared part way through the study were replaced by a new individual of the same species found closest to the original web. We observed each spider web in two rounds over three time periods—morning, afternoon, and evening—for a total of six rounds per day during 7 days. In this manner, webs were observed approximately every 1.5–3 hr throughout the day. Start time of observations was staggered each day to get an even estimate of prey capture throughout the 24‐hr cycle. A total of 78 webs from the 11 species were observed ranging from 2 to 15 webs per species (orbs = 30, tangle = 22, and sheet‐tangle = 26).

We considered “prey” to be insects or other arthropods that were caught in the webs and visibly handled or consumed by the spiders. Prey items were identified to the lowest possible taxonomic rank—morphospecies within Order or Family level. The body length (mm) of each prey item was estimated, using a caliper, from the anterior tip of the head to the posterior end of the abdomen. Regression equations for each Order or Family, from Sage (1982), were used to convert body length to dry mass (mg) for each prey type in order to estimate the body mass (hereafter, body size) of individual prey items (mg/dry mass) and also their total biomass (i.e., sum of the numerical abundance of a prey type times its body size). We did not measure dry mass of the prey directly because some of these individuals were partially eaten by spiders, which would have affected body mass estimates. In larger webs containing multiple prey items, such as those of *Anelosimus* species*,* the prey was removed after observation to avoid multiple counts of the same prey item. Because spider webs also vary in size, which can influence prey capture, web size was estimated by measuring the prey capture surface area of each web using a meter tape. Orb webs are relatively circular and their surface area was estimated as (π × *r* ^2^). Tangle webs were assumed to be approximately rectangular prisms, and their prey capture surface area was estimated as (2 × area of base + perimeter of base × height). The shape of sheet‐tangle webs was assumed to be a cone, and their prey capture surface area was estimated as (π × radius × slant height + π × radius^2^).

### 2.3 Sample collection

To estimate prey availability in the environment and conduct nutrient analyses, we collected separate insect and other arthropod samples from areas near the spider webs, immediately after the prey capture observations. We collected a total of 91 individual insects belonging to 31 morphospecies, 31 families, and 11 orders, with one to four representative individuals of each Order or Family. All prey individuals were identified to the lowest possible taxonomic rank. Immediately following the collection, prey were asphyxiated in a kill jar with ethyl acetate and, with their gut contents removed, dried for 48 hr in the laboratory oven at 60°C.

To determine the nutrient content of spiders, we collected samples from ten spider species (of 11 species included in the prey capture study) at the end of the study period. These samples comprised a total of 133 individuals, with four to eight individuals collected for each spider species, with the exception of the social *A. eximius* and *A. domingo* (social spiders live in large communal webs). For the latter two species, we collected two or three individuals from different sex and developmental stages (i.e., adult females, adult males, and subadult females) from eight *A. eximius* and five *A. domingo* nests. We did this to ensure that our chemical analyses captured the diversity of individuals within the colonies. Spiders were kept in the laboratory for 48 hr prior to asphyxiation in order to ensure gut contents were excreted (González et al., 2011). All spider samples were dried following the same procedure described for prey. No *Theridion* sp. individuals could be collected for stoichiometry analyses after the prey observation was concluded.

### 2.4 Sample preparation and arthropod chemical analysis

At the University of British Columbia (Vancouver, BC), we dried all arthropod samples a second time at 60°C for 48 hr prior to weighing to ensure there was no residual moisture. We determined dry mass (mg) of individuals (a proxy for body size) using a Mettler–Toledo electronic microbalance (±0.1 μg). For smaller arthropods (<0.4 mg dry mass), we performed carbon (C), nitrogen (N), and phosphorus (P) analyses on whole individuals. For larger arthropods, we analyzed C, N, and P contents in homogenized subsamples from dried individuals that were first crushed with a ball amalgamator (Henry Schein 101‐2691) or using a mortar and pestle. Tissue C and N content were measured with an elemental analyzer (Model Carlo Erba NC2500). Tissue C and N analyses were conducted at the Stable Isotope Laboratory at Cornell University, Ithaca, NY. We measured phosphorus (P) content in subsamples (~0.2 mg dry mass) of the homogenized arthropod individuals using potassium persulfate and sulfuric acid digestion followed by ascorbate–molybdate colorimetry (APHA, 1992). Percent recovery in P assays was estimated by comparison to apple leaf standards from the National Institute of Standards and Technology, US. (NIST‐1515). We describe “C, N, and P content” as the percentage of dry body mass. Nutrient ratios were calculated as molar ratios.

### 2.5 Statistical analyses

#### 2.5.1 Prey community analyses

To test whether spider web architecture influences prey capture, we did a set of four analyses based on: (1) richness of prey taxa captured (number of prey species captured by each spider type); (2) body size of prey captured; (3) prey community composition (i.e., the relative frequency and weighted biomass of prey types); and (4) the dispersion of the prey captured (frequency and weighted biomass) to assess spider's feeding mode as generalist or specialist (i.e., diet breath). The weighted biomass takes into account the average body mass of prey species *i*, the abundance of prey species *i*, and the number of prey species captured by the whole spider assemblage, providing information about the proportional contribution of each prey taxon to the spiders’ diet. For these analyses, we generated community matrices based on both prey frequency and biomass. The frequency‐community matrix summed the total frequency of each prey taxa observed in the webs of each spider species, resulting in a community matrix with 11 rows (one for each spider species) and 31 columns (one for each prey taxa). The biomass‐community matrix was the same as above except that we summed the total biomass (calculated as the number of individuals × body size) of each prey taxa observed in the webs of each spider species. To determine whether web types varied in the richness of prey taxa they captured, we performed individual‐based rarefaction (Gotelli & Colwell, 2001) on the frequency‐community matrix in order to account for unequal samples sizes of prey collected from our web observations. We then used ANOVA, followed by Tukey's HSD tests, to test for differences in prey species richness among web types, including web size as a covariate. To test whether web types differed in the composition of prey captured, we used separate permutational MANOVAs (PERMANOVA) for prey frequency and weighted biomass, respectively. PERMANOVA uses a permutation test to determine whether the observed community dissimilarities between web types are greater than the dissimilarities we would expect by chance. We chose the Morisita–Horn index to generate these dissimilarity matrices of the prey community composition, as this method is robust to unequal sample sizes and can handle biomass values (Barwell, Isaac, & Kunin, 2015; Krebs, 2013). To test for differences in prey dispersion (i.e., diet breath) between web types in terms of both frequency and weighted biomass, we used permutational analysis of dispersion followed by Tukey's HSD tests to determine specifically which groups differed. This test gives insight into the degree to which spider diets are specialized (low dispersion in prey types) versus generalized (large dispersion) in the prey community they capture. To visualize differences and variability in prey community composition, we used a canonical analysis of principal coordinates (CAP; Anderson & Willi, 2003), an effective ordination method for high‐dimensional datasets.

### 2.6 Stoichiometry of spider–prey interactions

Consumer–resource nutrient ratios are typically used as indicators of the strength of consumer nutrient limitation (Filipiak & Weiner, 2017; Matsumura et al., 2004; Sterner & Elser, 2002). Here, we used the threshold elemental ratio (hereafter TER, Sterner & Elser, 2002; Urabe & Watanabe, 1992) to identify the point at which a consumer (i.e., spider) growth switches from limitation by one element to another. Following Matsumura et al. (2004), for example, the C:N TER in a predator–prey interaction is given by:

$${\text{TER}}_{C:N}=\left(\text{C:N prey}/\text{C:N predator}\right)>\alpha_{N}/\alpha_{C}$$

where C:N prey and C:N predator are the C:N in prey and predator biomass, and α_N_ is the maximum gross growth efficiency for N (i.e., fraction of ingested N that the predator converts into new biomass), α_C_ is the maximum gross growth efficiency for C (i.e., fraction of ingested C that the predator converts into new biomass). To calculate the TER for each spider, we used a gross growth efficiency α_C_ = 0.65 C and α_N_ = 0.70 (Fagan & Denno, 2004; Fagan et al., 2002; Matsumura et al., 2004; Wiesenborn, 2013), and two values for α_PL_ = 0.6 (low maximum gross growth efficiency; Lehman, 1993) and α_PH_ (high maximum gross growth efficiency; DeMott, Gulati, & Siewertsen, 1998; Frost et al., 2006). Values for α_N_/α_C_ = 1.077, α_PL_/α_C_ = 0.923, α_PH_/α_C_ = 1.333, and α_PL_/α_N_ = 0.857, and α_PH_/α_N_ = 1.143. To estimate spider's TERs, we merged two datasets: (1) the dataset containing the elemental content data for multiple spider individuals of each species and web architecture; (2) the dataset on spider prey capture coupled to the stoichiometry data of the prey. The TER of each spider group was then used to determine the C:N, C:P, or N:P ratio of prey above which limited growth by N or P limitation may occur. The TER of each web‐building spider was tested for significance using *t* tests, which tells us whether C:nutrient prey/C:nutrient predator is significantly higher than the ratio of gross growth efficiencies.

Prior to testing the stoichiometry of spiders and their prey, we tested whether spiders with different web architectures differed in their body elemental content (C, N, P, C:N, C:P, and N:P) using general linear models (GLMs) with web type as the main factor and spider body size as a covariate. We then used generalized linear mixed‐effect models (GLMMs) to test whether biomass‐weighed stoichiometric interactions (N and P content) between spiders and their prey are elementally balanced as a function of web architecture. The models included %N, %P, or N:P ratio for individual spiders and their prey as the response variables and trophic level (i.e., predator or prey), web type, and body mass as fixed factors. Spider species was included as a random factor. This analysis took into account the biomass of the prey captured and its elemental content to reflect the relative elemental contribution of each prey type in the community to spider diets. If web type had a significant effect on the biomass‐weighed stoichiometric interactions between spiders and their prey, we conducted post hoc pairwise comparisons using Tukey's HSD tests. For these analyses, we considered mean ingestion efficiency for N of 68% and for P of 58% (Anderson & Hessen, 1995; Lehman, 1993). All statistical analyses were carried out using R v.3.3.0 (R Core Team, 2016).

## 3 RESULTS

### 3.1 Prey community analysis

**Figure 2:**
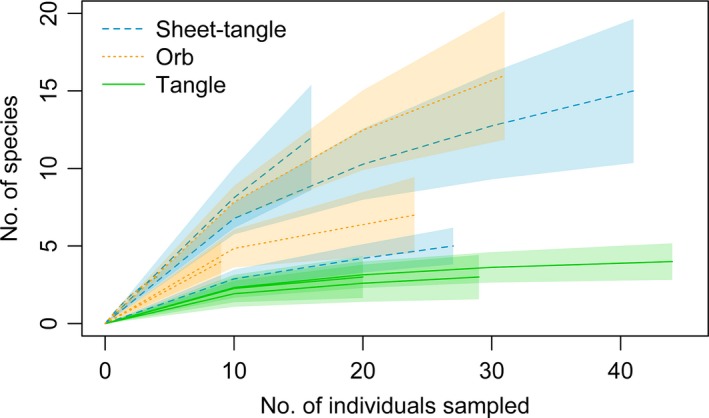
Rarefaction curves showing prey species accumulation with sampling effort (number of individuals sampled) for each spider species (11 species). Tangle webs (solid lines), which have lower prey richness, reach an asymptote quickly, while those with more diverse prey communities continue to accumulate greater prey richness with increasing sample size. Line types and colors correspond to different web architectures, and 95% confidence intervals for the rarefaction curves are shown

**Figure 3:**
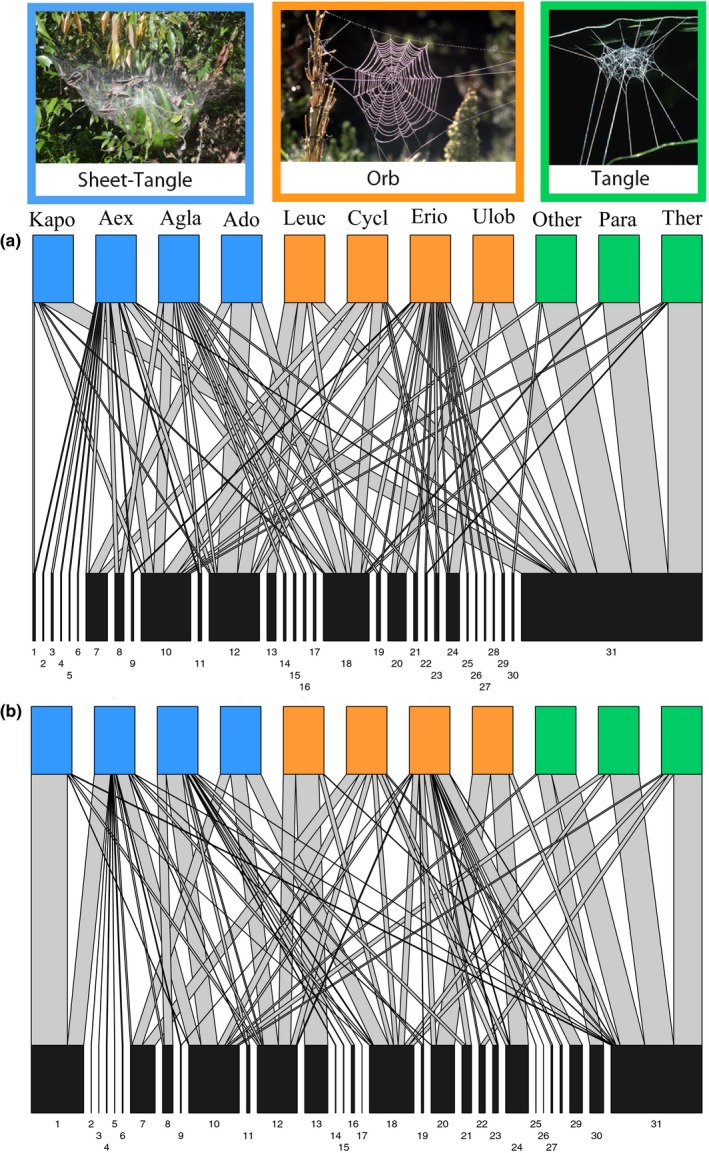
Bipartite food webs showing the observed spider–prey interactions in terms of the (a) the relative frequency and (b) the relative biomass of prey captured by each spider species. The top row contains all of the observed spider species (*n* = 11), colored by web type. The bottom row shows all prey types (*n* = 31). Gray lines connecting spider species to prey types show predatory interactions, with the width of the line representing the proportion of total capture for each spider species corresponding to a given prey type. The spider species are arranged in the network by web type. Spider species names associated with listed codes are as follow: (1) Kapo, *Kapogea sexnotata*; Aex, *Anelosimus eximius;* Agla, *Aglaoctenus castaneus*; Ado, *Anelosimus domingo*; Leuc, *Leucage* sp.; Cycl, *Cyclosa* sp.; Erio, *Eriophora* sp.; Ulob, Uloboridae sp.; Other (*Chrysso* sp., Theridiidae; *Architis* sp., Pisauridae), Para, *Parasteatoda* sp. and, Ther, *Theridion* sp. Prey types (Families or Orders) correspond to numbers as follows: (1) Acrididae sp.; (2) Apidae sp.; (3) Apocrita sp.; (4) Araneae sp.; (5) Berytidae sp.; (6) Blattaria sp.; (7) Carabidae; (8) Cercopidae sp.; (9) Chrysomelidae sp.; (10) Cicadellidae sp.; (11) Coccinellidae sp.; (12) Coleoptera other; (13) Curculionidae sp.; (14) Dermestidae sp.; (15) Tettigonidae sp.; (16) Formicidae sp.; (17) Fulgoridae sp.; (18) Gryllidae sp.; (19) Halictidae sp.; (20) Hemiptera sp.; (21) Histeridae sp.; (22) Isoptera sp.; (23) Lampyridae sp.; (24) Leiodidae sp.; (25) Lepidoptera sp.; (26) Membranicidae sp.; (27) *Micrathena* sp.; (28) Odonata sp.; (29) Reduviidae sp.; (30) Scarabaeidae sp.; and (31) Diptera sp.

**Figure 4:**
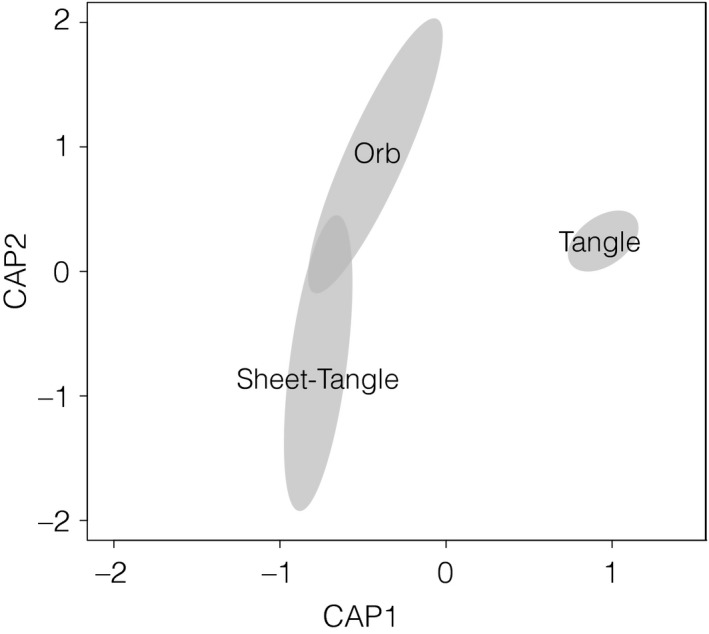
Ordination analysis using constrained analysis of principal coordinates, of dissimilarity in the composition of prey biomass captured by different types of web architectures. The position of the spider web types corresponds to the centroid ordination space, with the distance between centroids corresponding to the dissimilarity (Horn–Morisita index) in prey composition captured by different web types. The area of each gray ellipse represents the standard error of the centroid (*SE*) and thus variability in prey composition (i.e., diet breath). Note that tangle web spiders captured a distinct composition of prey biomass with little variability, indicating that they were specialists compared to orb‐web and sheet‐tangle spiders

Web type (*F*_2,7_ = 8.76, *p* = .010), but not web size (*F*_1,7_ = 2.93, *p* = .131), was an important predictor of prey richness (Figure 2). Likewise, web architecture (*F*_2,7_ = 5.81, *p* = .034) but not web size (*F*_1,7_ = 2.93, *p* = .309) affected prey size. For example, orb and sheet‐tangle web spiders captured twofold more prey taxa than tangle webs (tangle vs. orb, *p* = .012; tangle vs. sheet‐tangle, *p* = .020; Figure 2). Similarly, orb and sheet‐tangle webs captured prey that were, respectively, nine‐ and 29‐fold larger than prey captured by tangle webs (tangle vs. orb, *p* = .060; tangle vs. sheet‐tangle, *p* = .009). While we did not find a difference in the composition of prey captured by different web types (*F*_2,7_ = 2.12, *p* = .146; Figure 3a), we found that the composition of prey biomass captured by orb and sheet‐tangle webs was 90% dissimilar from that captured by tangle webs (*F*_2,7_ = 2.56, *p* = .006; Figure 3b). Specifically, it appeared that sheet‐tangle and orb web‐building species were all broad generalists, catching a wide variety of prey, whereas the tangle webs of the species in our study captured mostly flies (68% of relative biomass captured) (Figure 4). Although prey dispersion did not differ among webs types (*F*_2,7_ = 4.33, *p* = .053), we did find that the variability in the composition of prey biomass was higher for orb and sheet‐tangle webs, compared to tangle webs (*F*_2,7_ = 11.12, *p* = .003), lending further support to our observation that tangle webs were more specialized toward catching small flies.

### 3.2 Stoichiometry of spider–prey interactions

**Figure 5:**
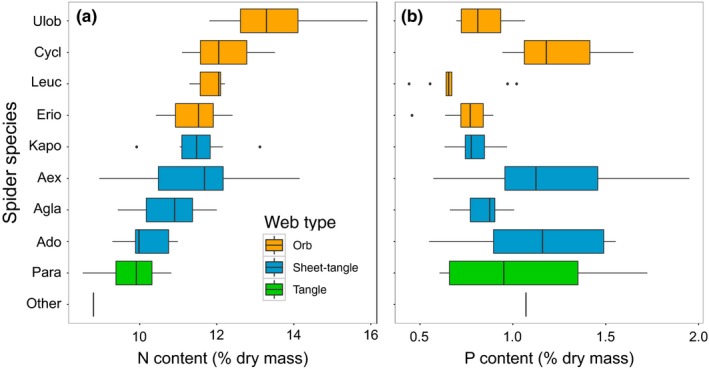
Nitrogen (a) and phosphorus (b) content of web‐building spiders. Spider species are grouped by type of web architecture they build; orb (orange), sheet‐tangle (blue), and tangle (green). Spider species names associated with listed codes are as follow: (1) Ulob, Uloboridae sp.; Cycl, *Cyclosa* sp.; Leuc, *Leucage* sp.; Erio, *Eriophora* sp.; Kapo, *Kapogea* sp.; Aex, *Anelosimus eximius;* Agla, *Aglaoctenus* sp.; Ado, *Anelosimus domingo*; Para, *Parasteatoda* sp.; and Other (*Chrysso* sp., Theridiidae; *Architis* sp., Pisauridae). We were not able to collect *Theridion* sp. individuals and thus do not have data on their nutrient content

**Figure 6:**
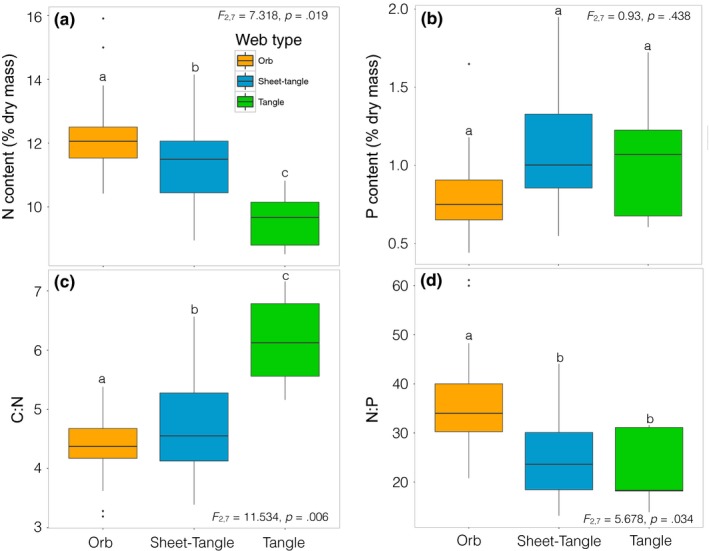
Boxplots of elemental contents of web‐building spiders for (a) N content, (b) P content, (c) C:N, and (d) N:P. Not shown, C and C:P. Boxes denote the interquartile range (25–75 percentile) containing the middle 50% of the data. The solid line is the median value. Whiskers denote the upper and lower extremes of the data (1.5 × IQR), and points represent statistical outliers. Different letters above the boxes indicate significant differences

Stoichiometric analysis revealed 1.5‐fold variation among spider species in N content and 1.8‐fold variation in P content (Figure 5). Spiders building different web architectures showed significant differences in their N, C:N, and N:P contents (Figure 6). Orb web spiders had higher N and N:P, but lower C:N than sheet‐tangle and tangle species, whereas sheet‐tangle spiders had higher N content and lower C:N than tangle web spiders (Figure 6). Spiders did not show significant differences in their C (*F*_2,7_ = 0.933, *p* = .438), P (Figure 6b), and C:P contents (*F*_2,7_ = 0.711, *p* = .524). Overall, the body size (range: 0.58–319 mg dry mass) of spiders did not have any significant effect on the elemental content variation among web‐building spiders (Table S1).

**Figure 7:**
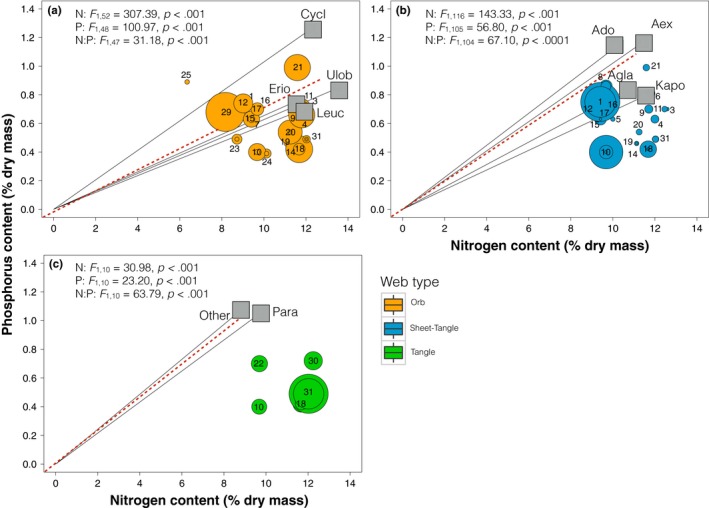
Bubble charts showing the average N and P content of spider species (squares) and their prey (circles) grouped by web type: (a) orb webs; (b) sheet‐tangle webs; and (c) tangle webs. The size of each prey bubble represents the proportional contribution of the prey type to the diet of the spider, determined by the prey biomass captured. The lines show the 1:1 N:P relationship between spiders and their prey community. The continuous black line represents the 1:1 relationship for each spider, whereas the dashed red line shows the 1:1 relationship for all spiders pooled (mean N and P contents). Bubbles overlapping the spider lines suggest that spiders and prey have the same N:P ratio. Bubbles below the line and toward the right side of the plot suggest that prey may have higher N:P than spiders, whereas bubbles above the line and toward the left of the plot suggest that prey may have lower N:P ratio than spiders. Codes for spider species and their prey as in Figure 3. The GLMMs take into account a mean ingestion efficiency for N of 68% and for P of 58% (Anderson & Hessen 1995; Lehman, 1993), the biomass of the prey captured and its elemental content to reflect the elemental relative contribution of each prey type in the community to spider diet (weighted analyses). We were not able to collect *Theridion* sp. individuals and thus do not have data on their nutrient content

The stoichiometry of spider–prey interactions showed a significant influence of web type on the elemental imbalances between spiders and their prey, and hence spider's TER. We found that sheet‐tangle web spiders display more balanced C:N interactions with their prey, while orb weavers, but particularly tangle spiders showed significantly high C:N imbalances (Table 1, Figure S1d). Overall, orb weavers and sheet‐tangle spiders captured prey of lower N content (and higher C content) than themselves; in contrast, tangle web spiders captured prey higher N content (and lower C content) than themselves (tangle spiders 9.59 ± 0.95 and prey 11.04 ± 1.18 N content). C:P and N:P imbalances were significant for all web‐building spiders (Table 1, Figure S1e,f), with higher P contents for all spiders than prey (Figure S1c). The mean TER_C:P_ for all web‐building spiders was c. 1.72 (α_PL_) and 1.29 (α_PH_) times higher than the mean body C:P, while the TER_N:P_ was c. 2.06 (α_PL_) and 1.54 (α_PH_) times higher than the mean body N:P, which are related to both N and P differences between spiders and their prey (Figure S1). Tangle web spiders displayed the largest C:P and N:P imbalances with their prey. When accounting for the biomass of captured prey, the stoichiometry of spider–prey interactions showed consistent N and P imbalances across web architectures (Figure 7). Both type of analyses yielded consistent results, and suggest nutrient limitation for web‐building spiders.

**Table 1:** Stoichiometric imbalances in spider–prey interactions tested against threshold elemental ratios of spiders (TER) calculated as, for example, TER_C:N_ = (C:N prey/C:N predator) > α_N_/α_C_, where C:N prey and C:N predator are the C:N in prey and predator biomass, and represent elemental imbalances, α_N_ is the maximum gross growth efficiency for N, and α_C_ is the maximum gross growth efficiency for C

Elemental ratio	Web architecture	*df*	*t*‐test	*p*‐value	α_N (P)_/α_C_	Stoichiometry prey/stoichiometry predator
C:N	Orb webs	29	2.2216	**.034**	1.077	1.165
	Tangle webs	6	−7.1926	**<.001**	1.077	0.727
	Sheet‐tangle	35	0.906	.814	1.077	1.049
C:P	Orb webs	29	5.946 (2.758)	**<.001 (<.01)**	0.923 (1.231)	1.498
	Tangle webs	6	6.563 (3.961)	**<.001 (<.01)**	0.923 (1.231)	1.699
	Sheet‐tangle	35	9.314 (4.849)	**<.001 (<.001)**	0.923 (1.231)	1.565
N:P	Orb webs	29	5.205 (1.921)	**<.001** (.065)	0.857 (1.143)	1.31
	Tangle webs	6	6.155 (5.048)	**<.001 (<.01)**	0.857 (1.143)	2.446
	Sheet‐tangle	35	8.296 (4.791)	**<.001 (<.001)**	0.857 (1.143)	1.534

Gross growth efficiency (GGE) was α_C_ = 0.65 C and α_N_ = 0.70, and two values for α_PL_ = 0.6 (low maximum GGE) and α_PH_ (high maximum GGE) shown in parentheses. Significant *p*‐values are highlighted in bold.

## 4 DISCUSSION

In this paper, we investigated how spider web architecture influenced the structure of spider–prey interactions on the basis of prey frequency and biomass as well as the stoichiometry of spider–prey interactions. Our results showed that web architecture partially affects prey richness and community composition. Only tangle webs showed a distinctive community based on prey richness and the relative biomass of the various prey types. Web‐building spiders tended to have higher N and P (and higher N:P) contents than their prey, except for tangle web spiders, which showed lower N content than their prey. The overall larger P imbalances (compared to N imbalances) between spiders and their prey suggest that P limitation of web‐building spiders may be of similar magnitude to N‐limitation described for other predators.

### 4.1 Prey community analyses

Among the three web architectures, tangle web spiders appear to be the only spiders that show any degree of prey specialization (Figures 2 and 3). Tangle webs had lower species richness and smaller dispersion between prey communities of the three web‐building groups. Furthermore, tangle webs had distinctive communities when the biomass of the prey was accounted for, with flies (dipterans) being the predominant prey type. These results contrast with previous studies showing that tangle web spiders tend to be more generalists than orb weavers and capture a similar range of resources as sheet‐tangle spiders (Sanders et al., 2015). Our findings likely reflect the importance of considering prey biomass when studying predator–prey interactions and resource generalization (see Blüthgen et al., 2006), as our analysis based on prey frequency, did not reveal prey differentiation among web types. This is probably because, even though tangle webs spiders captured mainly flies, the frequency with which other web types captured flies rendered the communities indistinguishable when only prey numbers were considered. In contrast, prey specialization was not evident in orb or sheet‐tangle webs, as they captured a high diversity of prey types, a similar composition based on the biomass of various prey types, and a large variability within species of each group. Aside from tangle webs, our results thus suggest little specialization as a function of web architecture. Literature supports that low prey specialization of orb web spiders and sheet‐tangle spiders seems to be a general pattern of web‐building spiders (Sanders et al., 2015; Wise & Barata, 1983). The large amount of prey variation among spider species within orb and sheet‐tangle types observed in our study (Figure 4) could also be indicative of intragroup variation in web architectures (e.g., web size, quality/thickness of the threads, location of the webs), which may have affected prey capture as well (Novak et al., 2010; Olive, 1980).

The size of captured prey differed among web types and this variation explains why tangle webs diverged from the other web types when the relative biomass of the prey was taken into account. As tangle‐web spiders only caught small prey, the relative proportions of the different prey types remained mostly unchanged when converted to biomass. In contrast, other web types captured a broader range of prey sizes. Although large prey are rarely caught, their large size reduces the contribution of smaller prey, such as flies, to the overall composition of the prey community in terms of relative biomass. Hence, the size of prey, but not its frequency, plays a key role in determining the actual relative biomass of each prey type captured by spiders (Blackledge & Eliason, 2007; Harmer et al., 2015). Web size has been shown to strongly correlate with mean prey size and prey size variation, such that larger spiders have shown larger differences in the composition of their diets when prey biomass is accounted for (Nentwig, 1982), although we did not observe any significant effect of web size on spider–prey interactions in our study.

Other important factors that may have influenced the difference in specialization in orb and sheet‐tangle webs are silk properties, web location, social structure, and even individual‐level differences within a spider species (Blackledge & Eliason, 2007; Opell, Bond, & Warner, 2006; Sanders et al., 2015; Watanabe, 1999, 2001). There are, for instance, two methods of creating sticky silk; cribellate spiders employ finely combed fibrils, whereas ecribellate spiders use a viscous glue‐like substance. These two methods may affect prey capture efficiency of different prey types (Opell, 1997, 1998). In fact, it has been shown that cribellate webs have improved retention of prey, which could enhance capture of larger or stronger prey types and minimize the size of a web without compromising function (Nentwig, 2013; Opell, 1994). In our study, we pooled together all orb web spiders; among these species; however, there was a cribellate spider (the uloboridae) with the remaining being ecribellate. Future work should include these (e.g., web architecture, size, orientation, location/elevation above ground, and silk properties) and other spider specific traits (e.g., venom properties and metabolic rates) that could affect the degree of specialization of spider–prey interactions. Similarly, social lifestyle and the construction of communal three‐dimensional webs by social spiders influence the insects captured with both sociality and colony size playing an important role in spider–prey interactions (Yip, Powers, & Avilés, 2008). In this study, half of the sheet‐tangle spiders had a social lifestyle while the other half were solitary spiders, and this could have increased variation in the prey types captured by spiders in this group.

### 4.2 Stoichiometry of spider–prey interactions

Web‐building spiders and their prey varied widely in their body nutrient content (Figures 5 and 6) and spider–prey interactions exhibited significant elemental imbalances (Figures 7 and S1). Our results showed that orb and sheet‐tangle spiders have significantly higher N content than their prey (but similar C content), suggesting potential N limitation for these species, as has been described for other predators (Fagan et al., 2002; González et al., 2011; Lemoine et al., 2014). In contrast, tangle web spiders, which appear to specialize on flies, have higher C:N contents (higher C and lower N contents) than either orb and sheet‐tangle web spiders (Figure 6), even though their prey (Figures 7 and S1) have similar N and C:N contents to the prey of other web‐building spiders. Our results for tangle‐web spiders contradict the general patterns observed for predators and prey, in which predators tend to have lower C:N than their prey because of their higher N and lower C contents (Fagan et al., 2002; González et al., 2011; Lemoine et al., 2014; Wilder, Norris, Lee, Raubenheimer, & Simpson, 2013; Woods et al., 2004). Our results suggest that this C imbalance for tangle‐web spiders may cause potential C limitation for this group of spiders. Spiders use extraoral digestion, which allows them to extract only edible nutrients and discard the inedible parts of their prey, such as wings and exoskeletons, which are high in N but especially in C (Wilder, 2011). The observation that tangle‐web spiders have higher C content than their prey provides support to the hypothesis that predators may suffer from C (i.e., energy) limitation (Wilder et al., 2013). Recent empirical research suggests that energy limitation may be common for some predatory species such as ants, beetles, and spiders (Grover, Kay, Monson, Marsh, & Holway, 2007; Kohl, Coogan, & Raubenheimer, 2015; Noreika, Madsen, Jensen, & Toft, 2016; Wilder et al., 2013; Wiggins & Wilder, 2018).

Although the structural and biochemical mechanisms underlying the high C and low N contents of tangle web spiders compared to other types of web‐building spiders are unknown, differences in lipid allocation to cuticule and in lipid stores to boost short‐ and long‐term metabolism may be likely explain the higher C content in tangle webs (Blackledge, Kuntner, & Agnarsson, 2011). Differences in the cuticular chemical composition, especially the lipid layer (C‐rich), vary with age, sex, and nutrition state affecting spider C content (Trabalon, 2013), whereas spider physiological state related to fat stores is also a main mechanism underlying intra‐ and interspecific differences in body C content (Lease & Wolf, 2011). It is also well known that food limitation (i.e., resource quantity) may select for higher nutrient storage in predators, especially energy dense molecules such as lipids to help fuel metabolism during periods of starvation (Hawley, Simpson, & Wilder, 2014; Jensen et al., 2012). Our results showed that tangle web spiders capture prey less frequently, and these prey have smaller body size than the prey captured by orb and sheet‐tangle spiders, which may induce larger carbon storage in tangle web spiders.

The stoichiometric analysis of spiders and their prey also revealed that spiders tend to have consistently higher P content than their prey, with overall larger P imbalances than N imbalances. Although P limitation in predator–prey interactions has been rarely studied, some studies have suggested that P may be similarly limiting (relative to N) for higher trophic levels (González et al., 2011; Lemoine et al., 2014). C:P imbalances were significantly higher than the TER_CP_ for all spiders, with the largest C:P imbalance for tangle web spiders. The main prey for tangle‐web spiders, dipterans, tends to have low P content (~0.50%P). Prey specialization by tangle web spiders seems to have resulted in larger P, C:P, and N:P imbalances for these spiders. In contrast, even when orb and sheet‐tangle web spiders showed significant elemental imbalances with their prey, these elemental mismatches were lower than those evidenced for tangle web spiders, except for C:N (and N) content. Overall, these results suggest that generalist spiders, such as orb and sheet‐tangle species, could overcome elemental imbalances by having a more varied diet than prey specialists (Denno & Fagan, 2003).

Whereas the N requirements of the spiders in our study were based on body elemental content, it is important to recall that silk production requires the allocation of large amounts of N (Craig, 2003), and therefore, web chemical content and web investment (i.e., silk amount) are a very likely factor influencing the N requirements of web‐building spiders (Savory, 1960; Vollrath, 2005) and other silk‐producing organisms, such as caddisflies (González, Romero, & Srivastava, 2014). Web‐building spiders show significant differences in the N content of silk, with tangle having 13.2%N, orbs 15.8%N, and sheet‐tangle 15.5%N in their webs (ALG, unpublished data). Furthermore, spiders invest different amounts of silk when building a functional web, with tangle < orb <<< sheet‐tangle (ALG, unpublished data). Overall, estimations of the amount of N required to build and maintain a functional web is complex due to either the chemical properties of the silk itself, differences in the quantities required to construct their webs, or due to the silk recycling through silk ingestion processes, as orb weavers do (Opell, 1998). Further research into the nutrient budgets associated with silk production and energetic web investment in tangle and other types of spider webs may be a key to understanding the nutritional and energetic requirements in spiders, and determining whether C, N, or P supply from their prey may be limiting for web‐building spider fitness.

Empirical studies and theoretical work have shown that the balance between food quality and nutrient demands by predators can have large effects on predator's fitness (Jensen et al., 2012; Laspoumaderes, Beatriz Modenutti, Elser, & Balseiro, 2015), influence secondary production (Hall, 2009; Hall, Shurin, Diehl, & Nisbet, 2007), and nutrient dynamics (Leroux, Hawlena, & Schmitz, 2012; Leroux & Schmitz, 2015). The growth and reproduction of predators can be strongly affected by prey quality, with higher reproductive output in terrestrial predators when feeding in high protein (i.e., high N) prey (Barry & Wilder, 2013; Simpson et al., 2015; Wilder, 2011, 2013; Wilder & Eubanks, 2010; Wilder et al., 2013). Some studies also suggest that nonprotein energy (carbohydrates and lipids) may play an important role in predator population dynamics (Simpson et al., 2015). Furthermore, recent research is beginning to show the strong effects that predators have on ecosystem‐level processes (Schmitz, Hawlena, & Trussell, 2010), and the consequences of elemental imbalances in predator–prey interactions on the ratios of elements recycled by predators (Munshaw, Palen, Courcelles, & Finlay, 2013). As shown in this study, stoichiometric differences between spiders and prey caused elemental imbalances in predator–prey interactions. Early work has shown strong stoichiometric mismatches in herbivore–plant interactions (Elser et al., 2000; Hall, 2009), and although elemental imbalances between predators and prey in both nitrogen and phosphorus have received far less attention, our findings suggest these mismatches may be common in nature. Furthermore, our findings are particularly important in light of evidence suggesting that generalist predators that have a broader range of prey could satisfy their optimal requirements for all nutrients simultaneously compared to specialist predators. Finally, although our findings provide good evidence of possible elemental imbalances in predator–prey interactions in terrestrial systems, this study lacked data on the consequences of these elemental imbalances for spider's fitness and their potential ecosystem‐level impacts. Future research addressing the fitness consequences of elemental imbalances in predator–prey interactions will improve our understanding of how stoichiometric effects might affect food web structure.

## CONFLICT OF INTEREST

None declared.

## AUTHOR CONTRIBUTIONS

ALG conceived the idea for the study and collected field data; ALG and LL performed analyses, and wrote the paper; MB contributed to the manuscript design, performed analyses, and reviewed the manuscript; LA contributed to the study design and reviewed the manuscript; and JG contributed to the study design, collected field data, and reviewed the manuscript. LL was an undergraduate student at the University of British Columbia; ALG and LA were her supervisors. All authors gave final approval before publication.

## DATA ACCESSIBILITY

The dataset supporting this article are available in Dryad https://doi.org/10.5061/dryad.p2t0t00.
