# Supplementary figures and images for: Caught in the web: Spider web architecture affects prey specialization and spider–prey stoichiometric relationships
Source: Ecol Evol. 2018 May 30;8(13):6449–62. doi: 10.1002/ece3.4028 (PMC6053566; doi:10.1002/ece3.4028)

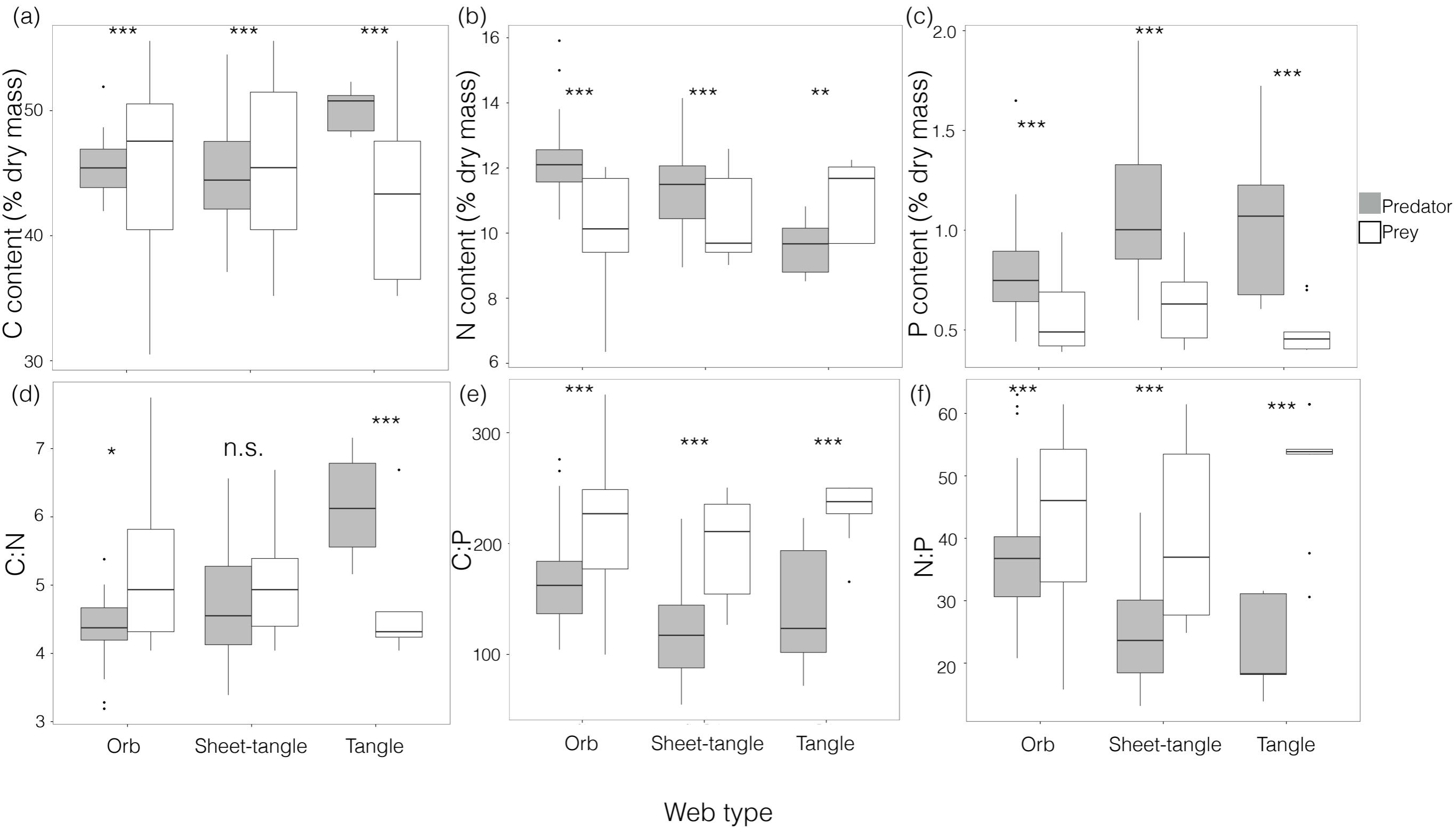

Supplement: Supplementary file 1 [file ECE3-8-6449-s001.pdf]
