# Supplementary material for: Caught in the web: Spider web architecture affects prey specialization and spider–prey stoichiometric relationships
Source: Ecol Evol. 2018 May 30;8(13):6449–62. doi: 10.1002/ece3.4028 (PMC6053566; doi:10.1002/ece3.4028)
Supplement: Supplementary file 2 [file ECE3-8-6449-s002.docx]

**Caught in the web: spider web architecture may affect prey specialization and spider-prey stoichiometric relationships**

Lorraine Ludwig^1^, Matthew Barbour^1^, Jennifer Guevara^2^, Leticia Avilés^1^ and Angélica L. González^3*^

^1^ Biodiversity Research Centre & Department of Zoology. University of British Columbia. BC. Canada

^2^ Universidad Regional Amazónica IKIAM. Vía Muyuna, Parroquia Muyuna. Tena, Napo, Ecuador.

^3^ Department of Biology & Center for Computational and Integrative Biology, Rutgers University. Camden, NJ. US.

*Corresponding author: A.L. González, 200 Federal street, Waterfront Technology Building, Camden, NJ. USA. E-mail address: [angelica.gonzalez@rutgers.edu](mailto:angelica.gonzalez@rutgers.edu)

**Online Supporting Information**

Table S1. Body size effect on the elemental content of web-building spiders.

| Element or ratio | df | F-value | *P*-value |
| --- | --- | --- | --- |
| C | 1,108 | 1.08 | 0.31 |
| N | 1,108 | 2.406 | 0.124 |
| P | 1,93 | 2.167 | 0.145 |
| C:N | 1,108 | 3.122 | 0.08 |
| C:P | 1,91 | 1.632 | 0.205 |
| N:P | 1,91 | 0.599 | 0.441 |

**Figure S1.** Nutrient content of spiders and prey grouped by web architecture for: (a) C content; (b) N content; (c) P content; (d) C:N; (e) C:P; and (f) N:P. Asterisks denote significant differences between means: * = *P* < 0.05, ** = *P* < 0.01, and *** = *P* < 0.001. n.s. = non-significant.

Figure S1.
